# Impact of Intranasal Administration of Fresh Breast Milk in Very Low Birth Weight Infants With Germinal Matrix-Intraventricular Hemorrhage

**DOI:** 10.7759/cureus.80416

**Published:** 2025-03-11

**Authors:** Gulay Sonmez Demir, Ozmert M Ozdemir, Musa Turgut, Yucel Pekal, Ece Koyuncu, Olcay Güngör, Hacer Ergin

**Affiliations:** 1 Department of Pediatrics, Division of Neonatology, Pamukkale University Faculty of Medicine, Denizli, TUR; 2 Department of Pediatrics, Division of Pediatric Neurology, Pamukkale University Faculty of Medicine, Denizli, TUR

**Keywords:** breast milk, intranasal, intraventricular hemorrhage, preterm babes, very low birth weight infants

## Abstract

There are no specific treatment modalities that prevent the progression of germinal matrix and intraventricular hemorrhage (GM-IVH) once it occurs. Breast milk is rich in mesenchymal stem cells, extracellular vesicles, and neurotrophins, all of which have been shown to have therapeutic effects. Intranasal drug administration has been demonstrated to be a reliable method for delivering various therapeutic agents directly to the brain via the olfactory nerve pathway. In this study, we aimed to evaluate the neuroprotective role of intranasal breast milk in very low birth weight (VLBW) infants with GM-IVH by assessing ventricular dilation and GM-IVH size on ultrasound, as well as neurodevelopmental outcomes using the Bayley-III scale.

This study included 22 VLBW infants diagnosed with GM-IVH in the first postnatal week. A total of 11 infants received intranasal breast milk (inFBM) for at least 28 days or until discharge, while 11 infants in the control group received standard treatment. Patients were clinically monitored via cranial transfontanelle ultrasonography. A total of 14 patients from both groups were evaluated using the Bayley-III Infant Developmental Screening Scale.

The incidence of posthemorrhagic ventricular dilation (PHVD) was significantly lower in the study group than in the control group (p = 0.010). In addition, the incidence of grade regression in the transfontanelle ultrasonography findings at discharge was significantly greater in the study group than in the control group (p = 0.008). Moreover, the incidence of retinopathy of prematurity was lower in the study group than in the control group.

Compared with non-administration, the intranasal administration of fresh breast milk to VLBW premature infants with GM-IVH results in regression of GM-IVH and significantly reduces progression to posthemorrhagic ventricular dilation. It may also reduce the incidence of retinopathy of prematurity.

## Introduction

With recent developments in the field of neonatal intensive care, the survival rates of babies with much lower gestational ages and/or birth weights have increased, but morbidities such as germinal matrix and intraventricular hemorrhage (GM-IVH) have emerged as more frequent problems. The occurrence of GM-IVH decreases as gestational maturity increases. Currently, its incidence is estimated to be approximately 20-25% in very preterm infants, whereas it affects nearly 45% of those born before 26 weeks of gestation [1]. The prevalence of GM-IVH among the very low birth weight (VLBW) infants in our neonatal intensive care unit (NICU) is approximately 27.5%.

GM-IVH is an important cause of brain damage, especially in extremely preterm infants, and continues to be a serious problem that adversely affects neurological development and increases mortality [1]. To the best of our knowledge, there is no specific treatment method other than supportive care, early detection and treatment of complications, early recognition of neurodevelopmental problems in the long term, and support of neuroplasticity through early intervention methods [1,2].

Breast milk is rich in growth factors, mesenchymal stem cells, exosomes, neurotrophins, and immune cells [3,4]. In experimental animal studies, neurotrophins and mesenchymal stem cells have been reported to be successfully delivered to the brain via nasal administration and to have therapeutic effects [5]. Exosomes, which are abundant in breast milk, especially colostrum, have therapeutic potential with various biological effects, including intercellular communication and signal transmission, coagulation, disease resistance, immune defense, cell proliferation, suppression of apoptosis, induction of angiogenic programs in endothelial cells, immunomodulatory signaling, programming of cells for tissue regeneration, and regulation of cell differentiation [6-8]. In healthy newborns, breast milk physiologically contacts the naso-oropharyngeal mucosa during breastfeeding. However, in the neonatal intensive care unit, this contact does not occur because very low birth weight infants are usually fed through an orogastric catheter.

In this study, we aimed to evaluate the neuroprotective role of intranasal fresh breast milk (inFBM) in very low birth weight infants with germinal matrix-intraventricular hemorrhage. Short-term neuromorphologic outcomes were assessed through serial transfontanelle ultrasonography (US) findings, focusing on ventricular dilation and GM-IVH size, while long-term neurodevelopmental outcomes were evaluated using the Bayley-III Infant Developmental Screening Scale [9].

## Materials and methods

This study included 22 infants born between September 2019 and May 2023 who were diagnosed with GM-IVH within the first week of life. Ethical committee approval was obtained from the Pamukkale University Non-Interventional Clinical Research Ethics Committee, with approval number E-60116787-020-324764. This study was conducted in accordance with the principles outlined in the Declaration of Helsinki. All infants included in the study were born at Pamukkale University Faculty of Medicine Hospital and received follow-up care and treatment in its tertiary neonatal intensive care unit (NICU).

The sample size was determined based on similar studies and established methodologies in the literature. The four-year study duration was planned considering the average annual number of eligible infants admitted to our NICU, the proportion of infants meeting the inclusion criteria, and the time required for follow-up assessments. Since the study focused on a defined patient population, it was projected that a sufficient number of participants could be recruited within four years. Additionally, since each infant was required to receive intranasal fresh breast milk for at least 28 days, the study timeline was structured to allow for adequate follow-up for all participants. The sample size was determined using statistical power analysis, ensuring that the minimum number of participants required to detect a clinically meaningful difference was included. However, due to the rarity of this patient population, we aimed to include as many eligible infants as possible within the study period.

Informed consent was obtained from the parents of all infants participating in the study. Standard neonatal resuscitation protocols were followed for delivery room management. The infants were transferred from the delivery room to our NICU using a transport incubator and received routine cluster care. From the first day, all infants were started on minimal enteral feeding (via an orogastric tube) and/or buccal mucosa oral care. Infants who were not breastfed and were instead fed formula milk were excluded from the study. Each preterm infant underwent transfontanelle ultrasonography (US) for the first three days and weekly thereafter. The data were analyzed prospectively.

Infants classified as VLBW, with a birth weight of less than 1500 grams, were included in the study. Since our hospital serves as a regional center, some infants had mothers residing in other cities. These mothers were only able to provide expressed breast milk once or twice a week in frozen form and could not supply fresh breast milk. In contrast, infants whose mothers stayed at the hospital and expressed breast milk eight times a day during care hours, as well as those whose mothers stayed in nearby apartment hotels and could deliver freshly expressed breast milk at least four times a day within two hours of expression, were able to receive intranasal fresh breast milk. Additionally, infants with severe congenital anomalies, congenital heart disease, chromosomal abnormalities, those diagnosed with IVH who died before 28 days of life, and those exclusively fed with formula milk were excluded from the study. As a result, the study and control groups were formed spontaneously.

In accordance with the study by Keller et al. [10], we administered intranasal fresh breast milk (inFBM) to 11 VLBW infants in our NICU who were diagnosed with GM-IVH after obtaining informed parental consent, and this group was classified as inFBM (+). Infants with GM-IVH whose mothers could not provide fresh breast milk (within two hours) to the NICU and whose parents did not consent to inFBM treatment were not administered inFBM and received routine treatment and care. This group was classified as non-inFBM or inFBM (-). Fresh breast milk obtained from healthy mothers within the last two hours of expression was gently instilled into both nasal cavities using the needleless tip of an insulin syringe, 0.1 ml, 4-8 times a day during the infants’ care hours. Intranasal fresh breast milk administration was initiated on the day GM-IVH was detected and continued for at least 28 days or until discharge.

Cognitive, language, and motor domains were assessed using the Bayley-III Infant Developmental Screening Scale by a certified pediatric neurologist in 14 patients, aged 18-36 months, from both groups [9]. This assessment was conducted on seven infants from each group, as only these infants were available for long-term neurodevelopmental follow-up at a corrected age of 18 to 36 months. In the evaluation of the cognitive, language, and motor domains, a composite score of 85 or above for each domain was considered normal, a composite score between 70-84 was classified as mild/moderate neurodevelopmental delay, and a composite score below 70 was defined as severe neurodevelopmental delay. The remaining infants were not assessed due to follow-up, parental refusal, or medical conditions that prevented evaluation.

Patients were clinically monitored via standard cranial transfontanelle US evaluations performed once in the first three postnatal days, weekly, and then once every two weeks after the four-week mark. A radiologist, unaware of the inFBM administration, graded the transfontanelle US evaluations according to the IVH classification defined by Volpe [2]. Post-hemorrhagic ventricular dilatation (PHVD) was classified into three stages based on the severity of ventricular enlargement and the presence of clinical symptoms, according to the methods of Murphy et al. [11]. Mild PHVD is characterized by ventricular enlargement without any clinical signs of raised intracranial pressure, and close monitoring with serial ultrasounds is recommended. Moderate PHVD involves progressive ventricular dilation, raising concerns about increased pressure, though definitive symptoms may not be present; in such cases, continued monitoring and potential intervention may be required. Severe PHVD is marked by significant ventricular enlargement accompanied by clinical signs of increased intracranial pressure, such as bulging fontanelle, splaying of sutures, irritability, apnea, and bradycardia. In these cases, surgical interventions, including ventriculoperitoneal (VP) shunting, may be necessary to manage the condition effectively. Hydrocephalus was defined as a condition in which the cerebral ventricular system contains an excessive amount of cerebrospinal fluid, leading to increased pressure and dilatation, and signs of intracranial pressure syndrome (ICPS), including an increase in head circumference (generally accepted upper limit: 2 cm/week), a tense anterior fontanelle, separation in the sutures between skull bones, setting sun in the eyes, bradycardia, apnea, and the development of respiratory and nutritional disorders [1,11,12]. Regression was evaluated by comparing the grade of transfontanelle US findings at discharge with the grade at the time GM-IVH was first detected.

The infants were then compared based on their perinatal data (birth week, birth weight, sex, delivery type, APGAR score, duration of invasive mechanical ventilation, survival until discharge, necrotizing enterocolitis “NEC”, patent ductus arteriosus “PDA” treatment, retinopathy of prematurity “ROP”, bronchopulmonary dysplasia “BPD”, sepsis, seizure, time to switch to full enteral feeding, corrected week at discharge, and duration of hospital stay) and transfontanelle US findings (first bleeding findings on US, regression in US findings at discharge, those who survived without needing surgery, those who developed PHVD, and those who developed post-hemorrhagic hydrocephalus and underwent surgery). Then, the scores of the 14 patients who were evaluated with the Bayley-III Infant Developmental Screening Scale were compared.

Statistical analyses were conducted using the Statistical Package for the Social Sciences (SPSS version 22.0, IBM Corp., Armonk, NY). Data were presented as median (range), mean ± standard deviation, or frequencies and percentages. For comparisons between two groups, the Mann-Whitney U test was used for continuous variables, and Fisher’s exact test was used for categorical variables. A p-value of <0.05 was considered statistically significant.

## Results

A total of 22 VLBW infants with grades 1-4 GM-IVH were included in the study. Of these, 11 received fresh breast milk via the intranasal route in addition to orogastric feeding, while the remaining infants did not receive intranasal fresh breast milk. The perinatal data of these infants, organized according to whether they received intranasal fresh breast milk or not, are presented in Table 1. One infant in the intranasal fresh breast milk group died on postnatal Day 30 due to severe sepsis. Cranial ultrasound conducted on postnatal Day 1 revealed grade 2 intraventricular hemorrhage in this neonate, prompting the initiation of intranasal administration of fresh breast milk on Day 2. There was no statistically significant difference in demographic and clinical data between the study and control groups.

**Table 1:** Demographic and clinical data of the groups receiving and not receiving intranasal breast milk inFBM(+): Those who received intranasal fresh breast milk; inFBM(-): Those who did not receive intranasal fresh breast milk, SD: standard deviation, C/S: cesarean section, NEC: necrotizing enterocolitis, PDA: patent ductus arteriosus, ROP: retinopathy of prematurity, BPD: bronchopulmonary dysplasia ^*^Mann‒Whitney U test for continuous variables and Fisher's exact probability test for categorical variables All values are provided in n(%) unless specified otherwise.

Variables	inFBM (+); n=11	inFBM (-); n=11	p-value^*^
Birth weight (grams), mean ± SD	826 ± 219	839 ± 716	0.748
Gestational age (weeks), mean ± SD	25.5 ± 2.2	26.3 ± 1.6	0.300
Female	8 (73%)	8 (73%)	
Male	3 (27%)	3 (27%)	1.000
Antenatal steroid, full cure	7 (63.6%)	7 (63.6%)	0.766
Antenatal steroid, incomplete cure	2 (18.2%)	1 (9.1%)	0.766
Antenatal steroid, no cure	2 (18.2%)	3 (27.3%)	0.766
Method of delivery (C/S)	11 (100%)	9 (82%)	0.476
1st minute Apgar Score (median, min-max)	5 (1-7)	6 (3-7)	0.949
5th minute Apgar Score (median, min-max)	7 (5-9)	7 (5-9)	0.438
Number of patients living until discharge (percent)	10 (90.9%)	11 (100%)	1.000
Duration of invasive mechanical ventilation (days), mean ± SD	9.2 ± 9.8	13 ± 12.7	0.652
NEC	6 (54.5%)	7 (63.6%)	1.000
PDA therapy	6 (54.5%)	7 (63.6%)	1.000
ROP	4 (36.4%)	9 (81.8%)	0.080
BPD	9 (66.7%)	8 (77.8%)	1.000
Sepsis	10 (90.9%)	11 (100%)	1.000
Seizure	6 (54.5%)	4 (36.4%)	0.670
Time to switch to full enteral feeding days (median, min-max)	26 (8-54)	19 (12-45)	0.63
Corrected age at discharge, weeks (median, min-max)	39 (36-46)	38 (35-41)	0.35
Duration of hospital stay(days), mean ± SD	74.3 ± 29.5	84.3 ± 14.5	0.365

The progress of the transfontanelle ultrasonography (US) findings, short-term results, and Bayley-III Infant Developmental Screening Scale-3 scores are summarized in Table 2. Comparisons of the severity of GM-IVH findings on the first day revealed no statistically significant differences between the two groups. The incidence of post-hemorrhagic ventricular dilatation (PHVD) was significantly lower in the study group (receiving intranasal breast milk) than in the control group (p= 0.010). Additionally, the incidence of grade regression in the transfontanelle US findings at discharge was significantly greater in the study group than in the control group (p= 0.008). There were no statistically significant differences between the two groups in other parameters (e.g., those who survived without needing surgery and those who developed posthemorrhagic hydrocephalus and required surgery). Bayley-III Infant Developmental Screening Scale scores were calculated for seven patients in the study group and seven patients in the control group, with no statistically significant difference found between the two groups. Although not statistically significant, the incidence of retinopathy of prematurity (ROP) was lower in the study group.

**Table 2:** Ultrasound findings, follow-up and Bayley-III evaluation of the cases. inFBM(+): Those who received intranasal fresh breast milk; inFBM(-): Those who did not receive intranasal fresh breast milk, TFUS: transfontanelle ultrasonography *Fisher's exact probability test for categorical variables.

Variables		inFBM (+)	inFBM (-)	p-value^*^
TFUS initial finding	Grade 1	3 (27.3%)	2 (18.2%)	0.940
	Grade 2	5 (45.5%)	5 (45.5%)	
	Grade 3	2 (18.2%)	3 (27.3%)	
	Grade 4	1 (9.1%)	1 (9.1%)	
Regression in TFUS findings at discharge	Yes	10(77.8%)	3 (27.3%)	0.008
	No	1 (22.2%)	8 (72.7%)	
Posthemorrhagic ventricular dilatation	Developing	3 (27.3%)	9 (81.8%)	0.010
	Not developing	8 (72.7%)	2 (18.2%)	
Those who develop posthemorrhagic hydrocephalus and require surgery		2 (18.2%)	1 (9.1%)	1.000
Bayley-III Infant Development Screening Scale		7 (63.6%)	7 (63.6%)	0.264
Those who survive and do not need any surgery		10 (90.9%)	9 (81.8%)	1.000

## Discussion

There are no specific treatment modalities that have been shown to prevent the progression of germinal matrix and intraventricular hemorrhage (GM-IVH) once it occurs. To the best of our knowledge, our approach after the occurrence of GM-IVH is based on the principles of supportive treatment, early detection and management of complications, early recognition of long-term neurodevelopmental issues, and promoting neuroplasticity with early intervention methods. Preventive measures, such as preventing preterm births, late cord clamping, avoiding hypothermia, hypoxia/hyperoxia, hypocarbia/hypercarbia, and hypotension/hypertension, as well as minimizing practices like off-label erythrocyte transfusion, and limiting physical contact with the infant during the first 72 postnatal hours while maintaining the head in a neutral position, are crucial [1,2].

Stem cells with multipotent and pluripotent properties found in breast milk account for 1-30% of the total breast milk cells [3,13]. In experimental animal models of neonatal diseases such as intraventricular hemorrhage, bronchopulmonary dysplasia, and necrotizing enterocolitis, stem cells have shown to be beneficial, with anti-inflammatory, immunomodulatory, anti-apoptotic, antioxidative, angiogenic, antifibrotic, and pro-regenerative effects [14,15]. Stem cells exert their effects through engraftment and extracellular vesicle (exosome secretome)-mediated paracrine mechanisms [7,16]. Extracellular vesicles, found in all body fluids (including breast milk, urine, blood, and cerebrospinal fluid), are involved in processes like intercellular communication, cellular homeostasis, and the transfer of functional biomolecules [17-20]. Milk-derived extracellular vesicles have been shown to enhance the viability, proliferation, and barrier function of intestinal epithelial cells, reduce apoptosis and intestinal damage, alleviate inflammation, and stimulate stem cell activity [21,22]. Breast milk also contains numerous neurotrophic factors, such as brain-derived neurotrophic factor, glial-derived neurotrophic factor, nerve growth factor, insulin-like growth factor-1, and hepatocyte growth factor [23]. Neurotrophins, even at nano-molar concentrations, strongly support the development, growth, and survival of neurons [24,25]. Moreover, observational studies on preterm infants indicate that breastfeeding in the neonatal intensive care unit significantly improves long-term neurocognitive outcomes [26].

Intranasal drug administration has been shown to be a reliable method for delivering therapeutic agents, such as growth factors and stem cells, directly to the brain via the olfactory nerve pathway [27]. In neonatal animal models, neuroprotective and regenerative effects, such as oligodendroglial growth, neuron growth, reduced damage, and improved neurologic outcomes, have been observed with intranasal administration of neurotrophins and mesenchymal stem cells [28,29]. Olfactory neurons, which serve as entryways to the brain and are exposed to the external environment, are selective not only for neurotrophins and stem cells but also for pathogens, such as amoebas, which also use the nasal route to reach the brain [30]. It has been reported that breast milk is rich in neurotrophins, which can cross the blood-brain barrier when administered intranasally [27-29]. Considering these findings, along with the study by Keller et al. [10], the first involving human neonates in this field, we administered fresh breast milk obtained from healthy mothers intranasally within two hours of expression. We evaluated the neuromorphologic outcomes through serial cranial ultrasounds and compared the validity of Keller et al.’s study with our own group of patients (grades I-IV GM-IVH). Short-term outcomes were assessed via serial transfontanelle ultrasound findings, and long-term neurological outcomes were assessed using the Bayley-III Infant Developmental Screening Scale-3 in VLBW infants with GM-IVH who received intranasal fresh breast milk (inFBM) versus those who did not (non-inFBM).

In a clinical study by Keller et al. [10], the frequency of porencephalic cyst formation was 21% in infants with Grade III and Grade IV IVH who received intranasal fresh breast milk, compared to 58% in those who did not receive it. The incidence of ventricular dilatation (71% vs. 91%) and post-hemorrhagic hydrocephalus requiring surgery (50% vs. 67%) was lower in the intranasal breast milk group, emphasizing that intranasal breast milk may be neurodevelopmentally beneficial in premature infants. In our study, we found that the frequency of ventricular dilatation was significantly lower in the intranasal breast milk group than in the non-intranasal breast milk group (27.3% vs. 81.8%, p = 0.010). We also observed a significant regression of GM-IVH in the intranasal breast milk group compared to the non-intranasal group (77.8% vs. 27.3%, p = 0.008) in the transfontanelle ultrasound performed at discharge, based on the initial bleeding findings. The frequency of post-hemorrhagic hydrocephalus requiring surgery was similar between the two groups.

No significant difference was found between the Bayley-III Infant Developmental Screening Scale scores. This lack of significance is likely due to the small number of patients available for testing. The Bayley-III assessment was performed on seven infants from each group, as only these infants were available for long-term neurodevelopmental follow-up between 18 and 36 months of corrected age. The remaining infants were not assessed for reasons such as loss of follow-up, parental refusal, or medical conditions that prevented evaluation. We acknowledge that the small sample size for the Bayley-III analysis is a limitation of our study. The reduced number of infants included may limit the statistical power and generalizability of the findings. We recognize this limitation and emphasize the need for larger, long-term follow-up studies to more effectively assess the neurodevelopmental outcomes of intranasal fresh breast milk administration in VLBW infants with GM-IVH. Additionally, although not statistically significant, the incidence of retinopathy of prematurity (ROP) was lower in the study group than in the control group, warranting further investigation in larger studies.

The main limitations of our study are the small number of patients, the evaluation of only short-term results, and the inadequacy of long-term data due to insufficient follow-up. Nevertheless, as this is the second study on this subject in the literature, and given the favorable short-term outcomes observed, we consider these results to be important.

## Conclusions

Compared with nonadministration, the intranasal administration of fresh breast milk to VLBW premature infants with GM-IVH results in regression of GM-IVH and significantly reduces the progression to posthemorrhagic ventricular dilatation. It may also reduce the incidence of ROP. However, additional large-scale randomized controlled trials with longer-term outcomes are needed for this to become a routine practice.
